# The Role of the Host Ubiquitin System in Promoting Replication of Emergent Viruses

**DOI:** 10.3390/v13030369

**Published:** 2021-02-26

**Authors:** Karl M. Valerdi, Adam Hage, Sarah van Tol, Ricardo Rajsbaum, Maria I. Giraldo

**Affiliations:** 1 Department of Microbiology and Immunology, University of Texas Medical Branch, Galveston, TX 77555, USA; kmvalerd@UTMB.EDU (K.M.V.); arhage@UTMB.EDU (A.H.); savantol@UTMB.EDU (S.v.T.); rirajsba@UTMB.EDU (R.R.); 2 Institute for Human Infections and Immunity, University of Texas Medical Branch, Galveston, TX 77555, USA

**Keywords:** ubiquitin system, emergent viruses, pro-viral function, antagonism of immune response, SARS-CoV-2, Ebola, Zika, Nipah, tripartite motif (TRIM) proteins

## Abstract

Ubiquitination of proteins is a post-translational modification process with many different cellular functions, including protein stability, immune signaling, antiviral functions and virus replication. While ubiquitination of viral proteins can be used by the host as a defense mechanism by destroying the incoming pathogen, viruses have adapted to take advantage of this cellular process. The ubiquitin system can be hijacked by viruses to enhance various steps of the replication cycle and increase pathogenesis. Emerging viruses, including severe acute respiratory syndrome coronavirus 2 (SARS-CoV-2), flaviviruses like Zika and dengue, as well as highly pathogenic viruses like Ebola and Nipah, have the ability to directly use the ubiquitination process to enhance their viral-replication cycle, and evade immune responses. Some of these mechanisms are conserved among different virus families, especially early during virus entry, providing an opportunity to develop broad-spectrum antivirals. Here, we discuss the mechanisms used by emergent viruses to exploit the host ubiquitin system, with the main focus on the role of ubiquitin in enhancing virus replication.

## 1. Introduction

**Figure 1:**
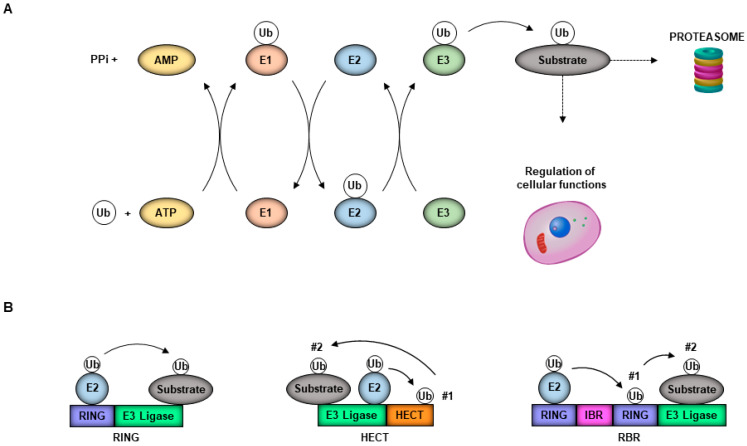
The ubiquitin system. (**A**) Free ubiquitin (Ub) is attached to a substrate after a series of ATP-dependent enzymatic reactions involving an E1-activating, E2-conjugating and E3-ligating enzyme. Substrates marked with Ub may be bound for the proteasome for degradation or can have activities altered to promote non-degradative cellular functions. (**B**) E3 ligases promote Ub transfer differently with RING family members, facilitating the movement of Ub directly from the E2 conjugase to the substrate, while HECT and RBR members first receive Ub from the E2 to their catalytic domain before delivering to the substrate.

Ubiquitin is a 76-amino acid protein well known for its function in marking proteins for degradation by the proteasome, although multiple non-degradative functions are well established. Covalent ubiquitination depends on a series of enzymes, including the E1 ubiquitin-activating enzyme, an E2 ubiquitin-conjugating enzyme, and an E3 ubiquitin ligase [1]. The E1 ubiquitin-activating enzyme activates ubiquitin in an ATP-dependent fashion [2]. The E2 ubiquitin-conjugating enzyme then forms a complex with the E1. Once the E1 is charged with ubiquitin, the ubiquitin is then transferred to a Cystine on the active site of the E2 [3]. The complex can then interact with one of three different classes of E3 ubiquitin ligases, the HECT, RBR or RING domain ligases; these then bring in a specific substrate. The HECT and RBR E3 ligases can transfer Ub directly to the substrate via formation of an ubiquitin–thioester intermediary on one of the E3 cysteine residues, while RING-containing E3 ligases position the Ub-loaded E2 in proximity to the substrate for E2-to-subtrate transfer, which usually occurs on a lysine (K) on that substrate (Figure 1A,B) [3]. The E3 ubiquitin ligases are interesting because they are the most diverse of the three enzymes, with more than 600 different E3 ligases [4]. Ubiquitin itself has seven lysines, each of which can also be conjugated to another ubiquitin to form a polyubiquitin chain. The E2 conjugase is an important determinant of the type of polyubiquitin chain that can be formed (specifically K6-, K11-, K27-, K29-, K33-, K48- or K63-linked), and the chain linkage is also important for the specific function. Both the E2 and the E3 ligases can be expressed in a cell-type or tissue-specific manner, or their expression can be induced in response to a specific stimulus [4], making the process of ubiquitination a complex process to study. The tripartite motif (TRIM) superfamily of E3 ligases is of particular interest for its roles in regulating the innate immune response and having direct and indirect antiviral activities, and enhancing virus replication [5].

Viruses have evolved to antagonize the host immune response by interfering with host ubiquitin-dependent signaling pathways, or by hijacking the cellular ubiquitination machinery, to promote viral replication and pathogenesis. With the ongoing pandemic caused by the novel strain of the severe acute respiratory syndrome coronavirus 2 (SARS-CoV-2), it is becoming critical to identify common steps used by different viruses to replicate in host cells that could potentially be targeted with broad-spectrum antivirals. Specifically, ubiquitination of viral proteins is emerging as an important host cellular process that is utilized by multiple viruses to promote their replication. In this review, we explore the various ways that emergent viruses exploit the host ubiquitin system, and we focus on recent literature highlighting the role of ubiquitin in enhancing virus replication (Table 1).

## 2. Ubiquitin System and Virus Entry

**Figure 2:**
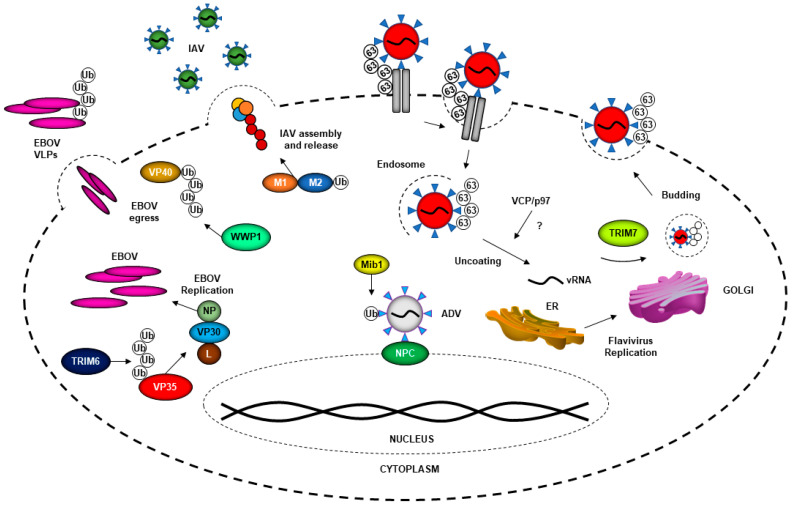
The host ubiquitin system can be hijacked by different viruses at different stages of their life cycles. Entry for flaviviruses like Zika virus (ZIKV) can be enhanced by ubiquitination of the envelope protein. The K63-linked ubiquitin ZIKV brings on its envelope protein allows for greater binding affinity to host receptors, improved cellular entry and higher titers. Adenovirus (ADV) hijacks the E3 ubiquitin ligase Mind bomb-1 (Mib1) to facilitate viral genome uncoating and release at the nuclear pore complex (NPC). Ebola virus (EBOV) can hijack TRIM6 to ubiquitinate VP35 to promote its polymerase cofactor activity and enhance viral replication. Egress of EBOV viral-like particles (VLPs) is enhanced by ubiquitination of EBOV VP40 protein by WWP1 (WW Domain Containing E3 Ubiquitin Protein Ligase 1). The influenza A virus (IAV) M2 protein can be ubiquitinated, allowing for better association with M1 and ultimately improved IAV assembly, budding and release of progeny virions.

Viruses gain entry to the host cell through highly specific interactions with cellular surface receptors, although some viruses can also use less-specific mechanisms of entry via interactions with lipids or carbohydrates on the surface of the cell membrane [27]. Following attachment, enveloped viruses undergo fusion with the cell at either the cell surface or endosomal membrane (Figure 2) [28]. The exact process of entry differs across viral families, and some viruses have used ubiquitin to enhance this process. This step is critical for productive virus infection and could provide a common target to inhibit multiple viruses. Indeed, many studies have investigated the use of proteasome inhibitors as potential therapeutics for viral infection. It has been reported that for human immunodeficiency virus (HIV), human astrovirus, herpes simplex virus, SARS and other viruses, the use of proteasome inhibitors negatively affects viral replication in vitro [29,30,31,32]. The proteasome can be used in a proviral fashion; however, the use of proteasome inhibitors is also known to deplete free cellular ubiquitin, which could also play a role in affecting viral replication and other functions that are important to maintain cellular homeostasis [33]. The proteasome inhibitor MG132 was found to inhibit mouse hepatitis virus (MHV), a murine coronavirus, at an early entry step, by promoting accumulation of viral RNA in the endosome and potentially inhibiting its release to the cytoplasm [34]. An example of a virus that hijacks the ubiquitination process in a proteasome-dependent manner to promote entry into cells is the flavivirus Japanese encephalitis virus (JEV) [6]. Proteasome inhibitors did not affect JEV attachment to the cells, or viral RNA translation, but did inhibit internalization at the level of releasing viral ribonucleoproteins (RNPs) from the endosome [6]. Additionally, small interfering RNA (siRNA) used to knock down ubiquitin in HeLa cells suggested that reduction in ubiquitin levels negatively impacted JEV entry; however, whether these effects were direct or indirect is unclear [6]. These results partially resemble observations made with a different flavivirus, dengue virus (DENV). When the activity of the E1-ubiquitin-activating enzyme UBA1 was inhibited with the UBEI 41 (Pyr-41) compound, uncoating of DENV was blocked [7]. Although the DENV capsid is degraded by the proteasome, inhibition of the proteasome does not affect virus uncoating. In this case, inhibition of the ubiquitination process by blocking the E1-activating enzyme resulted in inhibition of translation of viral RNA genome, but not virus internalization. Therefore, a ubiquitination step may be necessary for vRNA uncoating [7].

Further evidence that flaviviruses in general may use a non-degradative ubiquitination step for uncoating of the virus genome comes from experiments with yellow fever virus (YFV). Similar to DENV, inhibition of the E1-activating enzyme also inhibited the initial round of YFV translation, and this required the function of the valosin-containing protein VCP/p97 [10], which is known to interact with and extract ubiquitinated proteins from protein complexes [35,36]. Although it is currently unknown which E3-ubiquitin ligase is involved in this process and which viral protein may be ubiquitinated early, these studies strongly support a non-degradative function of ubiquitination of a viral protein that most probably occurs at a post-fusion stage and is required for flavivirus uncoating. However, flaviviruses can also take advantage of the ubiquitination process in an indirect manner. For example, DENV uses the ubiquitination of T cell immunoglobulin mucin 1 (TIM-1) to mediate its entry into cells [8]. On the cytosolic side of TIM-1, two lysine residues (K338 and K346) are needed for TIM-1 ubiquitination [8]. When cells expressing TIM-1 mutants (K338R, K346R and K338/346R) were challenged with DENV, they internalized less virus than when wild type TIM-1 was expressed, indicating that ubiquitination of receptors can also influence DENV entry [8].

While the examples mentioned thus far involve mechanisms in which the intracellular ubiquitination process promotes flavivirus entry, there is evidence that flaviviruses may contain ubiquitinated viral proteins in the infectious virion that promote extracellular interactions with the receptors. For example, a lysine residue (K38) on the envelope (E) protein of Zika virus (ZIKV), which is conserved in other members of the Flaviviridae family (DENV2, West Nile virus (WNV) and YFV), plays a direct role in viral entry [11]. Using recombinant infectious mutant viruses, replication of the ZIKV K38R mutant was significantly attenuated, in a cell-type-specific manner [11]. The host E3-ubiquitin ligase TRIM7 promoted ubiquitination of ZIKV E, and virus replication was reduced, especially in brain and reproductive tissues of infected *Trim7^−/−^* animals. A proportion of infectious viral particles released during replication contained ubiquitinated E, and ubiquitination on the E-K38 residue provided the virion the ability to interact with at least one potential cellular receptor, TIM-1, enhancing virus entry, replication and pathogenesis. In this case, ubiquitination of E not only functions in the early steps of virus entry, but also provides a mechanism of tissue tropism [11]. Further evidence that ubiquitination of E promotes better virus attachment and subsequent virus replication came from neutralization experiments using a specific anti-K63-linked-polyubiqutin antibody, which could reduce virus attachment and replication in tissue culture and in vivo [11]. However, the subcellular location where E ubiquitination occurs and how ubiquitinated E is incorporated into the virion remains unknown. An additional ubiquitination unique to ZIKV was on residue K281 of the enveloped protein. Although data suggest that ubiquitination on the E-K281 site may affect a step between virus attachment and uncoating, the precise role of ubiquitination on the K281 site during viral entry remains unclear [11].

Flaviviruses are not the only virus family that can hijack ubiquitin to better enter the cell. Ubiquitination of M1 of influenza A virus (IAV), an orthomyxovirus, has been found to play a role in the release of the virus from the late endosome during entry [12,13]. Human lung adenocarcinoma epithelial cells (A549) treated with shRNA against the E3 ligase ITCH (HECT-type ubiquitin E3 ligase [37]) revealed that there was more viral RNA (vRNA) in the cytoplasm of ITCH knockdown cells, as compared to the control. This inversely correlated with the amount of vRNA in the nucleus, indicating the release of vRNA from endosomes and its transport to the nucleus was delayed [12]. Additional experiments indicated that M1 undergoes direct ubiquitination by ITCH ubiquitin ligase, implicating the role of ubiquitination of M1 in early stages of IAV replication and/or entry [12]. Interestingly, IAV may also use unanchored polyubiquitin chains, which are not covalently attached to any protein, and seemed to be packaged in the infectious virion, for entry and efficient uncoating (Figure 2) [38]. These free ubiquitin chains are recognized by HDAC6, which is a component of the host aggresome pathway [39,40]. Although it is still unclear how IAV packages these unanchored ubiquitin chains, which ubiquitin enzymes are involved in this process, and how this may affect other functions of unanchored ubiquitin, including the innate immune response, this represents additional evidence of multiple ways in which ubiquitin promotes virus internalization and early steps of the replication cycle [41].

Another virus that uses ubiquitin to facilitate entry into cells is adenovirus (ADV). Ubiquitin regulates ADV’s ability to release its genome at the nucleopore of infected cells [15]. It was reported that siRNA-mediated knockdown of the E3-ubiquitin ligase Mind bomb-1 (Mib1) significantly reduced the viral load of ADV infection in vitro, and there was no effect on the early stages of ADV entry [15]. It was also determined that Mib-1 was needed for viral uncoating and genome release (Figure 2) [15].

Ubiquitination and proteasome-dependent degradation of cellular proteins could also provide strategies to limit virus entry. For example, a drug called halofuginone was identified in a screen to induce TMPRSS2 proteasomal degradation via the E3 ubiquitin ligase complex DDB1-CUL4 associated factor DCAF1 [42]. TMPRSS2 is a serine protease that promotes SARS and SARS-CoV-2 entry by proteolytic cleavage of the coronavirus spike protein required for virus attachment to the cell [43]. Proteasome inhibitors have also been proposed to inhibit other steps of the SARS-CoV-2 replication cycle [44].

## 3. The Ubiquitin System in Promoting Virus Replication

After a virus enters the cell, the virus uses a combination of the host-cell machinery and newly synthetized viral proteins to replicate its viral genome. Viruses have been found to utilize ubiquitin to enhance replication (Figure 2). In several studies, the use of proteasome inhibitors has been shown to block IAV protein synthesis [45,46]. It was discovered that at late stages of the IAV replication cycle, the deubiquitinase (DUB) USP11 can regulate IAV infection in cell-based in vitro assays [46]. Knockdown of USP11 in 293T cells resulted in increased IAV viral titers, while USP11 overexpression decreased viral titers [46]. Based on cellular-localization experiments, USP11 localizes in the nucleus and affects virus replication. Using a catalytic-defective USP11, the authors determined that USP11’s DUB activity is required for IAV RNA replication [46], and that these effects were dependent on monoubiquitination of residue K184 of the viral nucleoprotein (NP) [46]. A mutant K184R remained ubiquitinated because there were multiple ubiquitination sites on NP found by mass spectrometry (MS/MS), and could potentially compensate for the loss of ubiquitination on K184R [47,48]. Primer extension assays showed that ubiquitination is associated with increased transcription, and replication by the IAV polymerase, thus enhancing gene expression during IAV infection [47].

Interestingly, ubiquitination of viral polymerase factors may be a more general mechanism of regulation of the virus RNA transcription and replication steps. For example, the highly pathogenic Ebola virus (EBOV; family *Filoviridae*) VP35 protein directly uses ubiquitin to facilitate VP35′s polymerase cofactor activity [16]. The host E3-ubiquitin ligase TRIM6 ubiquitinates VP35 at K309 [16]. Although the VP35 K309 ubiquitination site is located in the interferon antagonist domain of VP35, ubiquitination of this residue enhances EBOV replication, and the absence of TRIM6 reduces viral replication (Figure 2) [16]. These results are also supported by experiments using an EBOV minigenome reporter assay, in which overexpression of TRIM6 but not a catalytically inactive mutant (TRIM6-C15A) enhances minigenome luciferase activity [16]. Although the precise mechanism is still unknown, ubiquitination of VP35 may affect interactions with factors of the viral polymerase regulating the balance of virus transcription/replication.

Ubiquitination is also suggested to play a role at the step of virus RNA replication of some flaviviruses. NS1 of DENV is ubiquitinated with K48-linked polyubiquitin chains. The ubiquitination of NS1 K189 was shown to reduce the interaction of NS1 with another viral protein, NS4B [9], and could play a role in the formation of the replication complex during DENV infection. In addition, as mentioned above, proteasome inhibitors reduce the levels of ZIKV and DENV RNA during the late stage of the replication cycle [7,11], suggesting that ubiquitination and subsequent proteasomal degradation of a viral (or host) protein is necessary for efficient virus RNA transcription and/or replication.

## 4. The Ubiquitin System in Virus Assembly and Budding

The ubiquitin system can be hijacked by viruses, leading to ubiquitination of their viral proteins to allow for assembly and budding. The IAV M2 protein has been shown to play several important roles in the viral life cycle. This protein not only participates in acidification in the endosome to allow uncoating and entry of the virus [49], but it also participates in the late stages of infection, such as assembly, budding and virus release [50]. Recent reports have found that M2 is ubiquitinated and its ubiquitination plays a critical role in the late stage of the influenza virus life cycle [14]. A K78R mutation on M2 reduced its ability to interact with the M1 protein, which is important for the efficient incorporation of vRNP into progeny virions [14]. It was determined that the M2-K78R mutation does not affect IAV entry or replication, but it does affect the assembly or production of infectious virus particles (Figure 2) [14]. The precise mechanism and the specific interaction with the components of the ubiquitin system remain unclear. Lassa virus (LASV), an old-world (OW) arenavirus, is known to cause viral hemorrhagic fever. This virus encodes four proteins: nucleoprotein (NP), surface glycoprotein precursor (GPC), L polymerase and RING finger protein Z. The Z protein plays an important role in viral assembly by assisting in the integration of GP, NP and polymerase L in viral progeny [51]. Z protein interaction with the ITCH E3 ligase was required for LASV replication. This interaction was found to occur through Z protein’s PPXY late domain, as has been shown for a range of other viruses, including lymphocytic choriomeningitis virus (LCMV) [20], and did not require the ubiquitin-E3 ligase activity of ITCH. Furthermore, ITCH is important for the production of infectious LASV particles and necessary for the release of viral progeny [52]. In EBOV, the PPxY late-domain motif of VP40 interacts with the WW domain of ITCH. This interaction regulates the budding of EBOV virus-like particles (VLPs) [17]. EBOV VP40 also has been shown to be SUMOylated, and this may regulate the stability of VP40. It was also proposed that SUMO and possibly ubiquitin could be incorporated into the VLPs, but the functions were not elucidated [18]. A similar mechanism has been reported between EBOV VP40 and the E3 ligase WWP1, in which interactions between the two are required for VLP budding (Figure. 2) [53]. Nedd4 is another E3-ubiquitin ligase that belongs to the HECT-type ubiquitin E3 ligase family [37] and is important in budding for EBOV and marburg virus (MARV) [19,54]. Nedd4 is found in the perinuclear region and can be associated with lipid rafts at the cytoplasmic membrane [54,55]. The protein structure of Nedd4 consists of a C2 membrane-binding domain, four central WW domains that bind to adaptors that generally contain PY motifs (PPxY or LPxY x is any residue) in target proteins [19] and a C-terminus homologous to the E6-AP carboxyl terminus (HECT) ubiquitin ligase domain [56]. Budding of EBOV and MARV VLPs in the presence of Nedd4 is perhaps modulated by Nedd4 ubiquitination, since there is a significant reduction when the HECT domain is mutated compared to wild-type Nedd4 [19,54].

LCMV has been increasingly recognized as a teratogen in recent years [57]. Nedd4 E3 Ub ligase is required for the release of LCMV particles, and this ubiquitination is used as a mechanism for the recruitment of ESCRT mediated by PPXY, an important complex for viral budding, suggesting that ubiquitination can be generated by other Z-associated proteins [20]. Paramyxoviruses can also utilize the ubiquitin system for the nuclear–cytoplasmic trafficking of the matrix protein (M). This was corroborated by mutations in the putative bipartite nuclear localization signal (NLS) and the leucine-rich nuclear export signal (NES) found in Nipah virus (NiV), Hendra virus (HeV), Sendai virus (SeV) and mumps virus [21], which prevented nuclear trafficking and budding. Through the ectopic expression of ubiquitin, an increase in budding was observed, but when proteasome inhibitors such as bortezomib were used, there was nuclear retention of M protein, and viral budding was blocked [21,58].

## 5. The Ubiquitin System and Viral Evasion of the Type-I Interferon (IFN-I) Response

**Figure 3:**
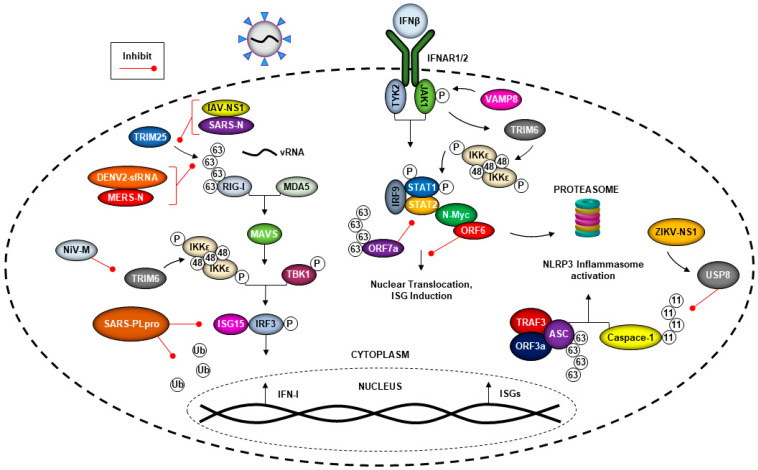
Viruses can evade immune responses utilizing the ubiquitin system. Viruses have evolved the ability to evade the host-innate immune response by antagonizing IFN production and signaling. SARS-CoV ORF6 can interact with N-Myc to promote its degradation through the ubiquitin proteasome, while ORF7a utilizes K63-linked polyubiquitin chains to prevent STAT2 phosphorylation and IFN-I signaling. The N protein of both SARS and MERS-CoV block the interaction between TRIM25 and RIG-I, preventing the K63-linked polyubiquitination of RIG-I needed for IFN-I production. TRIM25 is also a target of inhibition by the NS1 protein of IAV and the short noncoding sfRNAs of dengue-2 (DENV2). The PLpro component of SARS-CoV is capable of antagonizing innate immune pathways by acting as a deubiquitinase and by preventing the ISGylation of cellular proteins, including IRF3. Both SARS-CoV and Zika virus (ZIKV) alter components of the host ubiquitin system to activate the NLRP3 inflammasome, potentially leading to greater dissemination of viral progeny. The NS1 protein of ZIKV can recruit the deubiquitinase USP8 to cleave the K11-linked polyubiquitin chains from Caspase-1, while the SARS-CoV ORF3a protein promotes TRAF3 and ASC association, resulting in the K63-linked ubiquitination of ASC. In addition, ubiquitination also possesses antiviral functions. TRIM6 can regulate the expression of VAMP8 to promote JAK1 phosphorylation downstream of IFN-I signaling and promote the synthesis of unanchored K48-linked polyubiquitin chains for IKKε oligomerization.

The innate immune system is the first defense against pathogens and can detect virus invasion to limit virus replication. Innate immunity is activated when pattern recognition receptors (PRRs) that include Toll-like receptors (TLRs) and cytoplasmic RIG-I-like receptors (RLRs), recognize microbial components encoded in microorganisms that are known as pathogen-associated molecular patterns (PAMPs) [59]. Recognition of viral products by PRRs triggers multiple downstream signaling cascades via activation of kinases, including IκB kinases IKKα/β/ε, TBK1, TAK1 and others. Phosphorylation of multiple transcription factors including, but not limited to, IRF3, IRF7 and NF-κB, promotes their translocation to the nucleus, resulting in induction of IFN-I and proinflammatory cytokines [59]. RIG-I has an important role in virus RNA recognition and is activated upon ubiquitination by the E3-ubiquitin ligases TRIM25 and Riplet [60,61], and subsequent downstream signaling via the adaptor protein MAVS, which is also heavily regulated by the ubiquitination process, leading to IFN-I production [62]. Upon release from infected cells, IFNs are recognized by their receptor in an autocrine or paracrine manner and activate the Janus kinase signal transducer and activator of transcription (JAK-STAT) signaling pathway, leading to induction of antiviral host-effector proteins, called IFN-stimulated genes (ISGs) [63]. In addition, the E3-ubiquitin ligase TRIM6 regulates the phosphorylation and activation of IKKε-dependent STAT1 phosphorylation, and this can occur via VAMP8, a membrane protein associated with vesicles [64]. ISGs have a broad scope of antiviral mechanisms that are able to counter the viruses at different stages of their life cycles. Viruses have evolved multiple mechanism to block almost every step of these pathways. In many cases, this includes the inhibition of E3-ubiquitin ligases involved in the signaling, as discussed below (see Figure 3).

### 5.1. Coronaviruses Can Evade the Immune Response by Hijacking the Ubiquitin System

Coronaviruses have the ability to evade the host immune response, including antagonizing IFN production and signaling [65], which can lead to increased virus replication. The proteins encoded by the open reading frame 6 and 7a (ORF6, ORF7a), the nucleocapsid (N) and the papain-like protease (PLpro) proteins from both the previous epidemic strain of SARS-CoV and the new SARS-CoV-2 can antagonize the IFN response [23,24,66,67,68]. ORF6 has been the most studied for its structure and function. This protein has been shown to be highly conserved in SARS-related coronaviruses isolated from bats to humans [69]. SARS-CoV ORF6 can interact with Nmi (N-myc and STAT interactor) through the viral protein’s C-terminal 54 to 63 aa region, leading to N-Myc protein degradation through the ubiquitin proteasome pathway (Figure 3) [23]. On the other hand, the ORF7a protein of SARS-CoV-2 inhibits IFN-I signaling [24] via its K63-linked polyubiquitination on K119, which suppresses STAT2 phosphorylation [70]. Another factor of the ubiquitin system involved in IFN production that can be targeted by SARS-CoV is TRIM25. TRIM25 is well established as an E3 ligase that ubiquitinates RIG-I for downstream signaling to induce IFN-I [71]. The N protein of SARS-CoV can interact with the C-terminal SPRY region of TRIM25, blocking the interaction of TRIM25 with RIG-I, leading to inhibition of the signaling pathway (Figure 3) [67]. SARS-CoV N protein is essential in the virus-host interaction. Similarly, the N protein of Middle East respiratory syndrome CoV (MERS-CoV) can also interact with TRIM25 to inhibit RIG-I signaling [67]. Therefore, TRIM25 is a well-known target for IFN antagonism by multiple viruses, including well-known examples like the NS1 protein of IAV that inhibits RIG-I activation [72], or the subgenomic flavivirus RNA (sfRNA) of DENV-2, which binds TRIM25 to inhibit RIG-I activation and IFN expression [73].

Viruses can encode proteins with DUBs activity [32,44,74,75] that can enhance virus replication by both direct and indirect mechanisms. The PLpro protein of SARS-CoV is a DUB enzyme that is required for processing viral polyproteins to generate a functional replicase complex and enable viral spread [66]. This enzyme has the ability to cleave ubiquitin and ISG15, which is an ubiquitin-like molecule that is also a regulator of the innate immune response and has antiviral properties [76]. Some reports indicated that the inhibition of PLpro blocks SARS-CoV and SARS-CoV-2 replication [77,78]. The PLpro encoded by the previous epidemic strain of SARS-CoV reduced ubiquitinated substrates, and had a lower effect on ISGylated substrates, while SARS-CoV-2 PLpro reduced the presence of ISGylated proteins [79,80]. Upon infection with SARS-CoV-2, the PLpro protein promoted ISG15 cleavage from the transcription factor IRF3, inhibiting IFN-I induction [79,80]. Pharmacological inhibition of the ubiquitination SARS-CoV-2 proteins can reduce cytopathic effects while maintaining IFN-I responses and reducing virus replication [79,80], as well as potentially inhibiting self-processing of nsp3 to inhibit virus replication [81].

Ubiquitin plays a role in the activation of the NLRP3 inflammasome during infection by the previous epidemic strain of SARS-CoV-1, which could result in better dissemination of the virus in vivo [26]. SARS-CoV can induce both signals required for NLRP3 inflammasome activation, as it promoted the ubiquitination of P105, the transcription of pro-IL-1β genes and activation of NF-кB [26]. In addition, when ORF3a, TRAF3 and ASC associate with each other, it results in the K63-polyubiquitination of ASC and, subsequently, NLRP3 inflammasome activation [26]. This is an example of ubiquitin being used in a proviral fashion in an indirect manner (Figure 3).

### 5.2. Flavivirus Manipulation of the Ubiquitin System and the IFN Response

Interestingly, in addition to SARS, ZIKV has also been shown to induce activation of the NLRP3 inflammasome via its NS1 protein. This leads to an interesting mechanism of virus antagonism of the IFN response, by recruiting the DUB USP8, which cleaves the K11-linked polyubiquitin chains on caspase-1, blocking its degradation (Figure 3) [82]. This leads to increased cleavage of cGAS, which usually can detect mitochondrial DNA that leaks due to damage during infection [82,83,84], and results in IFN-I inhibition [82]. The NS1-mediated activation of the NLRP3 inflammasome attenuated IFN-I both in vitro and in vivo, and resulted in higher viral replication [82]. ZIKV and DENV also can antagonize IFN-I induction by cleaving cGAS and STING via their NS2B viral proteins [83,84,85]. Flaviviruses are also well known to inhibit IFN-I signaling via their NS5 proteins, and involve different mechanisms of ubiquitin-dependent targeting of STAT2. For in-depth information on the several studies on NS5-mediated inhibition of STAT2, please refer to recent excellent reviews [5,86,87,88,89,90,91,92].

### 5.3. Nipah Virus Matrix Protein (NiV-M) Inhibits TRIM6-Mediated IFN Responses

The host E3-ubiquitin ligase TRIM6, along with the E2-ubiquitin conjugase Ube2K, synthesize unanchored K48-linked polyubiquitin chains that enhance the activation and kinase activity of IKKε [93]. The unanchored ubiquitin promotes IKKε-dependent IFN-I induction through IRF3 phosphorylation to promotes IFN-I signaling through STAT1 phosphorylation at S708 [93]. The M protein of NiV, an emergent henipavirus, prevents K48 unanchored ubiquitin-mediated activation of IKKε through proteasome-independent TRIM6 degradation [22]. Due to the absence of TRIM6, the unanchored ubiquitin chains are not synthesized, and the IKKε-dependent roles in the IFN-I pathway are impaired [22]. Antagonism of IKKε-dependent IFN-I is conserved amongst the henipavirus M proteins, including hendra virus [22]. NiV-M’s degradation of TRIM6 and IFN-I antagonism requires the K258 residue, located in NiV-M’s nuclear-localization sequence [58], and the ability of M to localize to cytoplasmic membranes [22]. The mechanism underlying TRIM6′s degradation has not been identified, but NiV-M’s nuclear–cytoplasmic trafficking and ubiquitination could be required. Future experiments are also needed to investigate whether NiV-M can hijack TRIM6, similar to how EBOV VP35 exploits TRIM6 [16], to promote henipavirus replication (Figure 3).

Other henipaviral proteins block other steps of the IFN-I induction and signaling pathways. Similar to IAV and SARS- and MERS-CoVs, the accessory protein V (NiV-V) of NiV and other paramyxoviruses is capable of inhibiting TRIM25-dependent activation of RIG-I [25]. NiV-V interacts with both the SPRY domain of TRIM25 and the CARD domains of RIG-I [25]. The V protein is hypothesized to stabilize the TRIM25-RIG-I complex, and the stabilization of the complex impedes the downstream steps of the IFN-I induction pathway, including TRIM25-mediated ubiquitination of RIG-I [25].

## 6. Conclusions and Future Directions

To successfully replicate, viruses depend on specific factors expressed by the host cell. Many viruses use the ubiquitin system at different stages of their replication cycle, highlighting the importance of this cellular machinery for viral replication and spread. In this review, we provided an overview of the complexity of the interaction between emerging and highly pathogenic viruses such as SARS-CoV-2 and the ubiquitin system. Importantly, ubiquitination of structural proteins that are important in the process of virus entry is developing as a common non-degradative mechanism used by emergent viruses from different families, and could provide targets for broad-spectrum antivirals. One aspect that needs further investigation is how and when ubiquitinated proteins, or in some cases unanchored ubiquitin, are packaged in the infectious virions. Future studies should focus on identifying the specific steps and the subcellular compartments in which ubiquitination occurs to promote viral replication, and whether these processes are conserved through the different families of viruses. Identifying the specific factors of the host ubiquitin system that are commonly required for replication of different viruses could provide targets for broad-spectrum antivirals.

## Figures and Tables

**Table 1:** Comprehensive table of how emerging viruses mentioned in this review can utilize the ubiquitination of both host and viral proteins to enhance or complete their life cycles.

Virus	Target	Mechanism of Action	Reference
JEV	Unknown	Viral internalization, and release of viral RNPs from endosome.	[6]
DENV	Genome uncoating	Host E1 UBA1 implicated in vRNA uncoating and or translation.	[7]
DENV	Host TIM-1	Ubiquitination of TIM-1 at K338 and K346 increases virus internalization and is important for entry	[8]
DENV	NS1 protein	Ubiquitinated at NS1 K189, reduces interaction with viral NS4B	[9]
YFV	Genome uncoating	Post-fusion stage, viral uncoating	[10]
ZIKV	E protein	Ubiquitination of viral E protein at K38 drives viral entry and pathogenesis	[11]
IAV	M1 protein	Ubiquitinated by host ITCH ubiquitin ligase, affects release from endosome	[12,13]
IAV	M2 protein	Ubiquitination at K78 facilitates interaction with viral M1, which allows for efficient incorporation of vRNA into virion progeny	[14]
ADV	Unknown	Ubiquitination function of host Mib1 needed for viral uncoating, and genome release	[15]
EBOV	VP35 protein	Ubiquitinated at K309, enhances polymerase activity	[16]
EBOV	VP40 protein	VP40 interacts with ITCH and regulates the budding of EBOV virus-like particles (VLPs)	[17]
EBOV	VP40 protein	SUMOylated regulates the stability of VP40	[18]
EBOV	Unknown	Nedd4 monoubiquitination may modulate budding of VLPs	[19]
LCMV	Viral Z protein	Mechanism for recruitment of ESCRT, an important complex for viral budding	[20]
NiV, HeV, SeV, Mumps	Matrix protein	Nuclear–Cytoplasmic trafficking of the matrix protein.	[21]
NiV	M protein	Viral M protein prevents unanchored K48 Ubiquitin activation of IKKε through proteasome-independent TRIM6 degradation	[22]
SARS-CoV	ORF6 protein	ORF6 can interact with N-myc and STAT interactor, leading protein degradation through the ubiquitin proteasome pathway	[23]
SARS-CoV-2	ORF7a protein	K63 polyubiquitination on K119 suppresses STAT2 phosphorylation	[24]
SARS-CoV, MERS-CoV	Interference of RIG-I ubiquitination	Viral N protein can interact and block TRIM25 from ubiquitinating RIG-1	[25]
SARS-CoV	Host ASC	ORF3a, TRAF and ASC association results in K63 polyubiquitination of ASC and NLRP3 inflammasome activation	[26]

## Data Availability

Not applicable.
